# Supplementary figures and images for: A frequentist one-step model for a simple network meta-analysis of time-to-event data in presence of an effect modifier
Source: PLoS One. 2021 Nov 1;16(11):e0259121. doi: 10.1371/journal.pone.0259121 (PMC8559936; doi:10.1371/journal.pone.0259121)

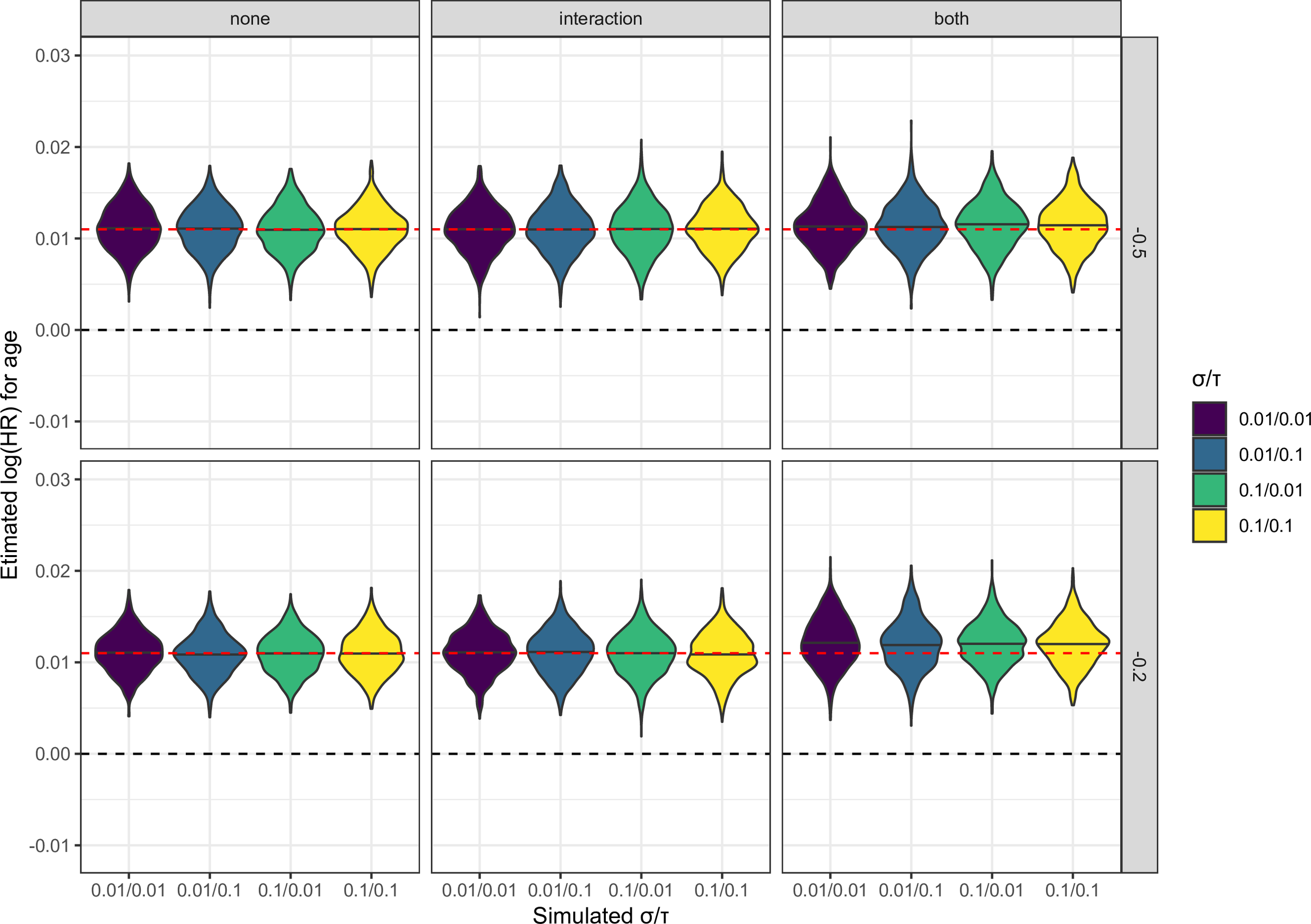

Supplement: S1 Fig — Distribution of estimated age effect in the Individual Patients’ Data based Poisson’s one step models according to the true treatment effect with log hazard ratio = -0.5 and -0.2 (rows) and the three scenarios none, interaction and both (columns) for configuration 1 Sigma (σ) represents the between-trial heterogeneity for the baseline risk and tau (τ) the between-trial heterogeneity of the treatment effect. Red dashed line represents the true effect of age.None: no interaction, same age distribution; Interaction: interaction in AC, same age distribution; Both: interaction in AC, different age distribution. (TIF) [file pone.0259121.s001.tif]

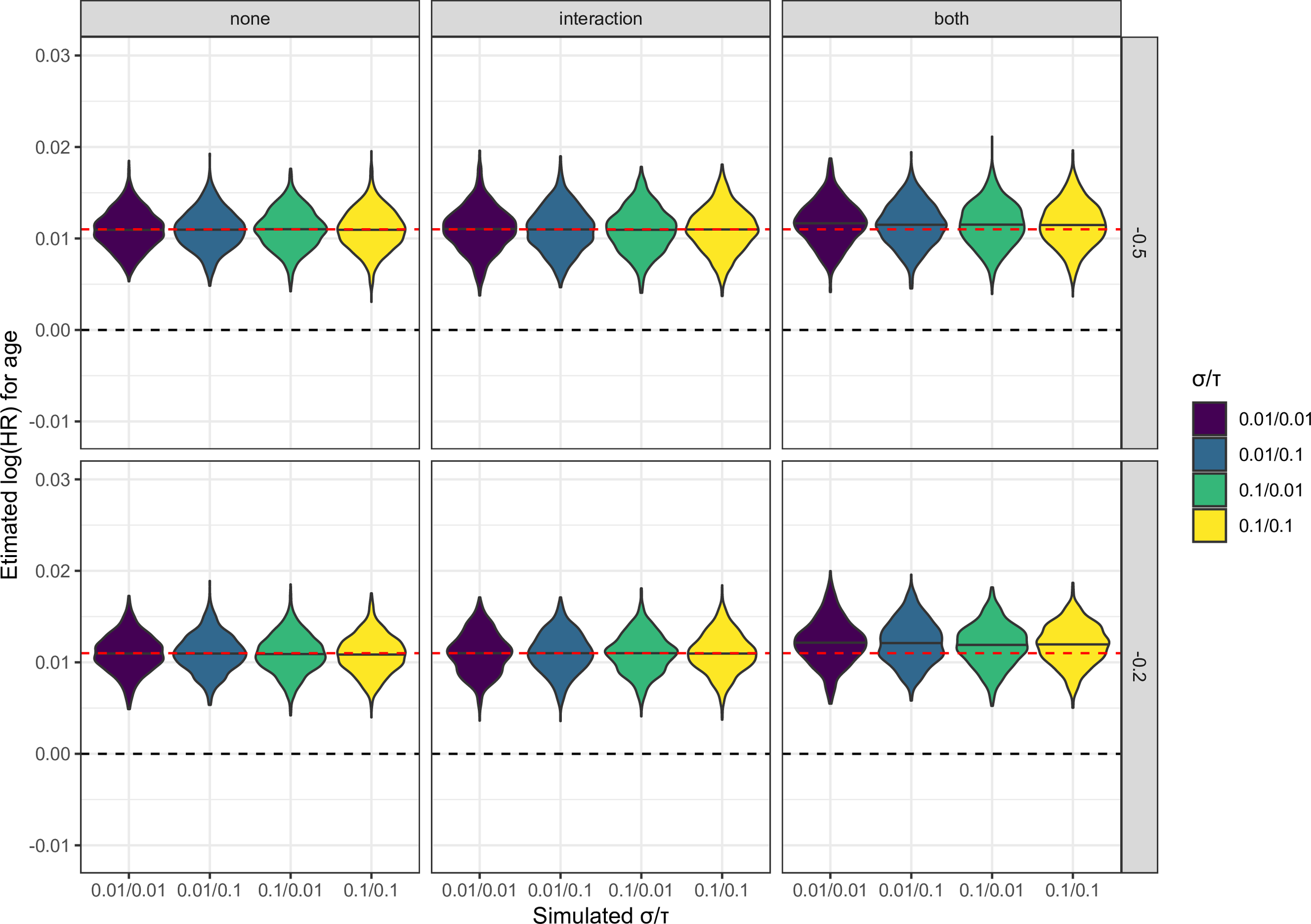

Supplement: S2 Fig — Distribution of estimated age effect in the Individual Patients’ Data based Poisson’s one step models according to the true treatment effect with log hazard ratio = -0.5 and -0.2 (rows) and the three scenarios none, interaction and both (columns) for configuration 2 Sigma (σ) represents the between-trial heterogeneity for the baseline risk and tau (τ) the between-trial heterogeneity of the treatment effect. Red dashed line represents the true effect of age. None: no interaction, same age distribution; Interaction: interaction in AC, same age distribution; Both: interaction in AC, different age distribution. (TIF) [file pone.0259121.s002.tif]
