# Supplementary material for: A frequentist one-step model for a simple network meta-analysis of time-to-event data in presence of an effect modifier
Source: PLoS One. 2021 Nov 1;16(11):e0259121. doi: 10.1371/journal.pone.0259121 (PMC8559936; doi:10.1371/journal.pone.0259121)
Supplement: S1 Table — Simulation results (Bias and Empirical Standard Error) of the A-C and B-C pairwise treatment comparisons in all simulation settings Both configuration are 3 nodes (A-B-C) network, with no closed loop in configuration 1 (no A-B trials) and a closed loop in configuration 2. IPD-Poisson is an Individual Patients’ Data model based on Poisson’s hierarchical model, AD-Metareg an aggregated-data based metaregression model an AD-Netmeta and aggregated-data model based on contrast. ttt: treatment effects as log(HR) with two possibilities: -0.2 and -0.5, σ: between-trial heterogeneity of baseline risk 0.01 or 0.1), τ: between-trial heterogeneity of treatment effect 0.01 or 0.1), S: scenario with three possibilities (1: no interaction, same age distribution; 2: interaction in AC, same age distribution; 3: interaction in AC, different age distribution), Param: parameters estimated by the model with age in years as a 4-class categorical variable (<55, 55–60, 60–65, >65) or as a continuous variable (TEage = 60: marginal effect = log(HR) for a patient of age 60; vTEage+1: the variation in log(HR) for a variation of one year of age), NA: Not applicable. (PDF) [file pone.0259121.s003.pdf]

S1 Table: Simulation results (Bias and Empirical Standard Error) of the A-C and B-C pairwise treatment comparisons in all simulation settings

					Configuration 1												Configuration 2											
					A-C						B-C						A-C						B-C					
					IPD Poisson1		AD metareg		AD-netmeta		IPD Poisson1		AD metareg		AD-netmeta		IPD Poisson1		AD metareg		AD-netmeta		IPD Poisson1		AD metareg		AD-netmeta	
ttt	σ	τ	s	param	Bias	ESE	Bias	ESE	Bias	ESE	Bias	ESE	Bias	ESE	Bias	ESE	Bias	ESE	Bias	ESE	Bias	ESE	Bias	ESE	Bias	ESE	Bias	ESE
-0.5	0.01	0.01	1	<55	-0.003	0.103	NA	NA	0.001	0.053	-0.007	0.104	NA	NA	0	0.052	-0.004	0.095	NA	NA	-0.002	0.047	0.001	0.095	NA	NA	0.001	0.046
-0.5	0.01	0.01	1	55-60	-0.001	0.108	NA	NA	0.001	0.053	0.006	0.106	NA	NA	0	0.052	-0.006	0.098	NA	NA	-0.002	0.047	-0.003	0.1	NA	NA	0.001	0.046
-0.5	0.01	0.01	1	60-65	-0.004	0.107	NA	NA	0.001	0.053	-0.004	0.11	NA	NA	0	0.052	0.001	0.094	NA	NA	-0.002	0.047	0.003	0.096	NA	NA	0.001	0.046
-0.5	0.01	0.01	1	>65	0.001	0.103	NA	NA	0.001	0.053	0	0.098	NA	NA	0	0.052	0	0.09	NA	NA	-0.002	0.047	0.002	0.087	NA	NA	0.001	0.046
-0.5	0.01	0.01	1	$TE_{age=60}$	-0.001	0.053	0.001	0.055	0.001	0.053	-0.001	0.052	0	0.057	0	0.052	-0.002	0.047	-0.001	0.05	-0.002	0.047	0.001	0.046	0.001	0.049	0.001	0.046
-0.5	0.01	0.01	1	vTE_{age+1}	0	0.007	0.004	0.109	0	0	0	0.006	-0.006	0.115	0	0	0	0.006	0.002	0.097	0	0	0	0.006	0.003	0.096	0	0
-0.5	0.01	0.01	2	<55	0.004	0.122	NA	NA	0.161	0.062	-0.003	0.107	NA	NA	0.001	0.055	0.002	0.105	NA	NA	0.16	0.053	0	0.095	NA	NA	0.003	0.048
-0.5	0.01	0.01	2	55-60	-0.003	0.125	NA	NA	0.044	0.062	-0.003	0.106	NA	NA	0.001	0.055	0.001	0.115	NA	NA	0.043	0.053	-0.004	0.1	NA	NA	0.003	0.048
-0.5	0.01	0.01	2	60-65	0.006	0.125	NA	NA	-0.031	0.062	0	0.109	NA	NA	0.001	0.055	0	0.111	NA	NA	-0.032	0.053	0.002	0.099	NA	NA	0.003	0.048
-0.5	0.01	0.01	2	>65	0.002	0.115	NA	NA	-0.148	0.062	0	0.104	NA	NA	0.001	0.055	0.006	0.097	NA	NA	-0.149	0.053	0.003	0.09	NA	NA	0.003	0.048
-0.5	0.01	0.01	2	$TE_{age=60}$	0.002	0.061	0.006	0.065	0.007	0.062	-0.001	0.054	0.001	0.058	0.001	0.055	0.002	0.053	0.005	0.056	0.006	0.053	0.001	0.047	0.003	0.05	0.003	0.048
-0.5	0.01	0.01	2	vTE_{age+1}	0	0.008	-0.002	0.126	-0.016	0	0	0.007	-0.005	0.114	0	0	0	0.007	-0.003	0.107	-0.016	0	0	0.006	0	0.102	0	0
-0.5	0.01	0.01	3	<55	-0.035	0.081	NA	NA	0.037	0.063	-0.004	0.242	NA	NA	0.003	0.052	-0.037	0.077	NA	NA	0.062	0.054	-0.028	0.157	NA	NA	-0.017	0.048
-0.5	0.01	0.01	3	55-60	-0.004	0.135	NA	NA	-0.08	0.063	0.002	0.169	NA	NA	0.003	0.052	-0.002	0.12	NA	NA	-0.054	0.054	0.009	0.137	NA	NA	-0.017	0.048
-0.5	0.01	0.01	3	60-65	-0.011	0.175	NA	NA	-0.155	0.063	-0.002	0.117	NA	NA	0.003	0.052	-0.005	0.146	NA	NA	-0.13	0.054	0	0.109	NA	NA	-0.017	0.048
-0.5	0.01	0.01	3	>65	-0.032	0.254	NA	NA	-0.272	0.063	0.002	0.065	NA	NA	0.003	0.052	-0.01	0.162	NA	NA	-0.246	0.054	0	0.063	NA	NA	-0.017	0.048
-0.5	0.01	0.01	3	$TE_{age=60}$	-0.002	0.083	-0.026	1.108	-0.117	0.063	0.001	0.074	0.016	0.871	0.003	0.052	0.001	0.066	0.041	0.543	-0.092	0.054	0.002	0.062	0.04	0.546	-0.017	0.048
-0.5	0.01	0.01	3	vTE_{age+1}	0	0.008	-0.004	0.138	-0.016	0	0	0.007	-0.002	0.109	0	0	0	0.006	0.004	0.068	-0.016	0	0	0.006	-0.005	0.068	0	0
-0.5	0.01	0.1	1	<55	-0.001	0.108	NA	NA	0.003	0.062	0.001	0.11	NA	NA	0.005	0.065	0.008	0.1	NA	NA	0.003	0.056	0.007	0.097	NA	NA	0.004	0.055
-0.5	0.01	0.1	1	55-60	0.001	0.109	NA	NA	0.003	0.062	0	0.111	NA	NA	0.005	0.065	0.001	0.102	NA	NA	0.003	0.056	0.003	0.104	NA	NA	0.004	0.055
-0.5	0.01	0.1	1	60-65	0.001	0.112	NA	NA	0.003	0.062	0.008	0.117	NA	NA	0.005	0.065	-0.002	0.101	NA	NA	0.003	0.056	0.005	0.1	NA	NA	0.004	0.055
-0.5	0.01	0.1	1	>65	0.001	0.109	NA	NA	0.003	0.062	0.002	0.108	NA	NA	0.005	0.065	-0.001	0.091	NA	NA	0.003	0.056	-0.003	0.094	NA	NA	0.004	0.055
-0.5	0.01	0.1	1	$TE_{age=60}$	0.001	0.061	0.004	0.066	0.003	0.062	0.003	0.064	0.003	0.069	0.005	0.065	0.002	0.056	0.004	0.057	0.003	0.056	0.003	0.055	0.003	0.057	0.004	0.055
-0.5	0.01	0.1	1	vTE_{age+1}	0	0.007	-0.005	0.129	0	0	0	0.007	0.003	0.131	0	0	0	0.006	0.002	0.111	0	0	0	0.006	-0.003	0.112	0	0
-0.5	0.01	0.1	2	<55	0.012	0.126	NA	NA	0.164	0.069	0	0.114	NA	NA	0.003	0.062	0.002	0.108	NA	NA	0.16	0.06	-0.001	0.102	NA	NA	0	0.055
-0.5	0.01	0.1	2	55-60	0.001	0.13	NA	NA	0.048	0.069	0	0.112	NA	NA	0.003	0.062	0.004	0.118	NA	NA	0.043	0.06	-0.005	0.105	NA	NA	0	0.055
-0.5	0.01	0.1	2	60-65	0	0.127	NA	NA	-0.028	0.069	0.006	0.113	NA	NA	0.003	0.062	0.001	0.112	NA	NA	-0.032	0.06	-0.002	0.101	NA	NA	0	0.055
-0.5	0.01	0.1	2	>65	0.007	0.115	NA	NA	-0.145	0.069	-0.006	0.107	NA	NA	0.003	0.062	0	0.103	NA	NA	-0.149	0.06	-0.004	0.093	NA	NA	0	0.055
-0.5	0.01	0.1	2	$TE_{age=60}$	0.005	0.07	0.009	0.074	0.01	0.069	0	0.061	0.002	0.066	0.003	0.062	0.002	0.061	0.005	0.065	0.005	0.06	-0.003	0.056	0	0.059	0	0.055
-0.5	0.01	0.1	2	vTE_{age+1}	0	0.007	-0.002	0.147	-0.016	0	0	0.007	0	0.13	0	0	0	0.007	0.003	0.122	-0.016	0	0	0.006	0.002	0.119	0	0
-0.5	0.01	0.1	3	<55	-0.038	0.085	NA	NA	0.036	0.068	-0.007	0.252	NA	NA	0.003	0.062	-0.03	0.085	NA	NA	0.065	0.063	-0.019	0.163	NA	NA	-0.018	0.056
-0.5	0.01	0.1	3	55-60	-0.008	0.139	NA	NA	-0.081	0.068	-0.001	0.168	NA	NA	0.003	0.062	-0.009	0.128	NA	NA	-0.052	0.063	-0.002	0.137	NA	NA	-0.018	0.056
-0.5	0.01	0.1	3	60-65	-0.005	0.178	NA	NA	-0.157	0.068	-0.004	0.123	NA	NA	0.003	0.062	-0.008	0.146	NA	NA	-0.128	0.063	0.003	0.114	NA	NA	-0.018	0.056
-0.5	0.01	0.1	3	>65	-0.029	0.256	NA	NA	-0.273	0.068	0.002	0.072	NA	NA	0.003	0.062	-0.001	0.155	NA	NA	-0.244	0.063	-0.001	0.07	NA	NA	-0.018	0.056
-0.5	0.01	0.1	3	$TE_{age=60}$	-0.001	0.087	-0.004	1.21	-0.119	0.068	-0.003	0.082	-0.033	1.044	0.003	0.062	0.003	0.071	-0.017	0.607	-0.09	0.063	0.002	0.067	-0.016	0.612	-0.018	0.056
-0.5	0.01	0.1	3	vTE_{age+1}	0	0.008	-0.001	0.151	-0.016	0	0	0.007	0.004	0.13	0	0	0	0.006	-0.003	0.076	-0.016	0	0	0.005	0.002	0.077	0	0

					Configuration 1												Configuration 2											
					A-C						B-C						A-C						B-C					
					IPD Poisson1		AD metareg		AD-netmeta		IPD Poisson1		AD metareg		AD-netmeta		IPD Poisson1		AD metareg		AD-netmeta		IPD Poisson1		AD metareg		AD-netmeta	
ttt	σ	τ	s	param	Bias	ESE	Bias	ESE	Bias	ESE	Bias	ESE	Bias	ESE	Bias	ESE	Bias	ESE	Bias	ESE	Bias	ESE	Bias	ESE	Bias	ESE	Bias	ESE
-0.5	0.1	0.01	1	<55	0.001	0.104	NA	NA	0.007	0.053	0.004	0.109	NA	NA	0.003	0.055	0.001	0.093	NA	NA	0.002	0.049	-0.002	0.088	NA	NA	0.001	0.047
-0.5	0.1	0.01	1	55-60	0.005	0.114	NA	NA	0.007	0.053	0.002	0.107	NA	NA	0.003	0.055	0.005	0.094	NA	NA	0.002	0.049	0.001	0.097	NA	NA	0.001	0.047
-0.5	0.1	0.01	1	60-65	0.007	0.112	NA	NA	0.007	0.053	-0.007	0.113	NA	NA	0.003	0.055	0.002	0.099	NA	NA	0.002	0.049	0.003	0.094	NA	NA	0.001	0.047
-0.5	0.1	0.01	1	>65	0.008	0.098	NA	NA	0.007	0.053	0.006	0.098	NA	NA	0.003	0.055	0	0.091	NA	NA	0.002	0.049	0.001	0.09	NA	NA	0.001	0.047
-0.5	0.1	0.01	1	$TE_{age=60}$	0.006	0.053	0.007	0.057	0.007	0.053	0.002	0.055	0.002	0.058	0.003	0.055	0.002	0.049	0.002	0.051	0.002	0.049	0.001	0.047	0.002	0.05	0.001	0.047
-0.5	0.1	0.01	1	vTE_{age+1}	0	0.007	-0.004	0.112	0	0	0	0.006	0.003	0.107	0	0	0	0.006	0	0.101	0	0	0	0.006	-0.004	0.101	0	0
-0.5	0.1	0.01	2	<55	0	0.121	NA	NA	0.159	0.06	0.002	0.107	NA	NA	0.002	0.051	0.007	0.105	NA	NA	0.158	0.054	0.007	0.095	NA	NA	0.003	0.049
-0.5	0.1	0.01	2	55-60	0.001	0.119	NA	NA	0.042	0.06	-0.003	0.113	NA	NA	0.002	0.051	-0.01	0.111	NA	NA	0.041	0.054	-0.003	0.102	NA	NA	0.003	0.049
-0.5	0.1	0.01	2	60-65	-0.002	0.125	NA	NA	-0.034	0.06	0.001	0.109	NA	NA	0.002	0.051	0.002	0.104	NA	NA	-0.034	0.054	0	0.1	NA	NA	0.003	0.049
-0.5	0.1	0.01	2	>65	0.001	0.114	NA	NA	-0.15	0.06	0.001	0.099	NA	NA	0.002	0.051	0.002	0.102	NA	NA	-0.151	0.054	-0.004	0.089	NA	NA	0.003	0.049
-0.5	0.1	0.01	2	$TE_{age=60}$	0	0.06	0.002	0.064	0.004	0.06	0.001	0.052	0.003	0.056	0.002	0.051	0.001	0.054	0.002	0.059	0.004	0.054	0	0.05	0.002	0.052	0.003	0.049
-0.5	0.1	0.01	2	vTE_{age+1}	0	0.008	-0.003	0.132	-0.016	0	0	0.007	0.003	0.117	0	0	0	0.007	-0.002	0.109	-0.016	0	0	0.006	0.002	0.098	0	0
-0.5	0.1	0.01	3	<55	-0.04	0.077	NA	NA	0.031	0.063	-0.015	0.244	NA	NA	0	0.054	-0.036	0.076	NA	NA	0.064	0.055	-0.018	0.163	NA	NA	-0.017	0.048
-0.5	0.1	0.01	3	55-60	-0.009	0.139	NA	NA	-0.085	0.063	0.001	0.156	NA	NA	0	0.054	-0.001	0.122	NA	NA	-0.052	0.055	0.002	0.131	NA	NA	-0.017	0.048
-0.5	0.1	0.01	3	60-65	-0.019	0.191	NA	NA	-0.161	0.063	0	0.123	NA	NA	0	0.054	-0.001	0.143	NA	NA	-0.128	0.055	0.002	0.106	NA	NA	-0.017	0.048
-0.5	0.1	0.01	3	>65	-0.034	0.262	NA	NA	-0.278	0.063	-0.001	0.067	NA	NA	0	0.054	-0.006	0.153	NA	NA	-0.244	0.055	0	0.065	NA	NA	-0.017	0.048
-0.5	0.1	0.01	3	$TE_{age=60}$	-0.005	0.086	0	1.057	-0.123	0.063	-0.002	0.074	-0.005	0.9	0	0.054	0.003	0.066	0.048	0.521	-0.09	0.055	0.002	0.063	0.048	0.522	-0.017	0.048
-0.5	0.1	0.01	3	vTE_{age+1}	0	0.008	0	0.132	-0.016	0	0	0.006	0.001	0.112	0	0	0	0.006	0.005	0.065	-0.016	0	0	0.006	-0.006	0.065	0	0
-0.5	0.1	0.1	1	<55	0.009	0.104	NA	NA	0.002	0.06	-0.005	0.108	NA	NA	0	0.061	0.005	0.096	NA	NA	0.004	0.057	0.002	0.099	NA	NA	0.004	0.056
-0.5	0.1	0.1	1	55-60	-0.003	0.116	NA	NA	0.002	0.06	-0.003	0.116	NA	NA	0	0.061	0.005	0.107	NA	NA	0.004	0.057	0.006	0.101	NA	NA	0.004	0.056
-0.5	0.1	0.1	1	60-65	-0.001	0.111	NA	NA	0.002	0.06	0.002	0.115	NA	NA	0	0.061	0.001	0.104	NA	NA	0.004	0.057	0.001	0.101	NA	NA	0.004	0.056
-0.5	0.1	0.1	1	>65	-0.002	0.102	NA	NA	0.002	0.06	-0.001	0.106	NA	NA	0	0.061	0.003	0.095	NA	NA	0.004	0.057	0.008	0.09	NA	NA	0.004	0.056
-0.5	0.1	0.1	1	$TE_{age=60}$	0.001	0.06	0.002	0.066	0.002	0.06	-0.002	0.061	-0.003	0.066	0	0.061	0.004	0.057	0.004	0.06	0.004	0.057	0.004	0.056	0.004	0.059	0.004	0.056
-0.5	0.1	0.1	1	vTE_{age+1}	0	0.006	0.005	0.127	0	0	0	0.007	0.002	0.129	0	0	0	0.006	-0.004	0.114	0	0	0	0.006	-0.003	0.109	0	0
-0.5	0.1	0.1	2	<55	0.004	0.128	NA	NA	0.159	0.069	0	0.109	NA	NA	0.004	0.061	0.003	0.112	NA	NA	0.163	0.062	0.002	0.095	NA	NA	0.004	0.055
-0.5	0.1	0.1	2	55-60	-0.003	0.13	NA	NA	0.042	0.069	-0.001	0.113	NA	NA	0.004	0.061	0.005	0.116	NA	NA	0.046	0.062	0.004	0.103	NA	NA	0.004	0.055
-0.5	0.1	0.1	2	60-65	0	0.122	NA	NA	-0.033	0.069	0.004	0.116	NA	NA	0.004	0.061	0.011	0.118	NA	NA	-0.03	0.062	0	0.107	NA	NA	0.004	0.055
-0.5	0.1	0.1	2	>65	0.002	0.118	NA	NA	-0.15	0.069	0.002	0.109	NA	NA	0.004	0.061	0.002	0.105	NA	NA	-0.146	0.062	0.001	0.094	NA	NA	0.004	0.055
-0.5	0.1	0.1	2	$TE_{age=60}$	0.001	0.069	0.003	0.074	0.004	0.069	0.001	0.061	0.004	0.066	0.004	0.061	0.005	0.062	0.008	0.065	0.008	0.062	0.002	0.055	0.004	0.058	0.004	0.055
-0.5	0.1	0.1	2	vTE_{age+1}	0	0.007	0.004	0.142	-0.016	0	0	0.007	-0.001	0.136	0	0	0	0.007	0	0.125	-0.016	0	0	0.006	0.003	0.114	0	0
-0.5	0.1	0.1	3	<55	-0.036	0.087	NA	NA	0.039	0.072	0.007	0.237	NA	NA	0.004	0.057	-0.035	0.082	NA	NA	0.066	0.062	-0.021	0.165	NA	NA	-0.015	0.056
-0.5	0.1	0.1	3	55-60	0.001	0.14	NA	NA	-0.077	0.072	0.003	0.164	NA	NA	0.004	0.057	0.009	0.122	NA	NA	-0.051	0.062	0.005	0.138	NA	NA	-0.015	0.056
-0.5	0.1	0.1	3	60-65	-0.003	0.19	NA	NA	-0.153	0.072	0.004	0.124	NA	NA	0.004	0.057	0.001	0.142	NA	NA	-0.126	0.062	0.002	0.114	NA	NA	-0.015	0.056
-0.5	0.1	0.1	3	>65	-0.004	0.251	NA	NA	-0.269	0.072	0.001	0.069	NA	NA	0.004	0.057	-0.002	0.161	NA	NA	-0.243	0.062	0.005	0.069	NA	NA	-0.015	0.056
-0.5	0.1	0.1	3	$TE_{age=60}$	0.009	0.092	0.022	1.16	-0.115	0.072	0.004	0.078	-0.038	1.066	0.004	0.057	0.007	0.072	-0.016	0.63	-0.088	0.062	0.002	0.069	-0.021	0.632	-0.015	0.056
-0.5	0.1	0.1	3	vTE_{age+1}	0	0.008	0.002	0.144	-0.016	0	0	0.006	0.005	0.133	0	0	0	0.006	-0.003	0.079	-0.016	0	0	0.006	0.003	0.079	0	0
-0.2	0.01	0.01	1	<55	0.002	0.099	NA	NA	-0.001	0.051	-0.004	0.1	NA	NA	0.001	0.051	0	0.092	NA	NA	0	0.046	-0.003	0.096	NA	NA	0.001	0.049
-0.2	0.01	0.01	1	55-60	-0.007	0.102	NA	NA	-0.001	0.051	-0.002	0.102	NA	NA	0.001	0.051	-0.004	0.091	NA	NA	0	0.046	0.003	0.097	NA	NA	0.001	0.049
-0.2	0.01	0.01	1	60-65	-0.002	0.104	NA	NA	-0.001	0.051	0.003	0.111	NA	NA	0.001	0.051	0.003	0.094	NA	NA	0	0.046	0.005	0.097	NA	NA	0.001	0.049
-0.2	0.01	0.01	1	>65	-0.001	0.098	NA	NA	-0.001	0.051	0.005	0.096	NA	NA	0.001	0.051	0.001	0.087	NA	NA	0	0.046	0.002	0.088	NA	NA	0.001	0.049
-0.2	0.01	0.01	1	$TE_{age=60}$	-0.002	0.051	-0.002	0.055	-0.001	0.051	0.001	0.05	0.001	0.055	0.001	0.051	0	0.046	0	0.049	0	0.046	0.001	0.049	0.002	0.051	0.001	0.049
-0.2	0.01	0.01	1	vTE_{age+1}	0	0.006	0.001	0.108	0	0	0	0.006	0	0.106	0	0	0	0.006	0.002	0.101	0	0	0	0.006	0.006	0.092	0	0

					Configuration 1												Configuration 2											
					A-C						B-C						A-C						B-C					
					IPD Poisson1		AD metareg		AD-netmeta		IPD Poisson1		AD metareg		AD-netmeta		IPD Poisson1		AD metareg		AD-netmeta		IPD Poisson1		AD metareg		AD-netmeta	
ttt	σ	τ	s	param	Bias	ESE	Bias	ESE	Bias	ESE	Bias	ESE	Bias	ESE	Bias	ESE	Bias	ESE	Bias	ESE	Bias	ESE	Bias	ESE	Bias	ESE	Bias	ESE
-0.2	0.01	0.01	2	<55	0.002	0.1	NA	NA	0.066	0.052	0	0.102	NA	NA	0	0.05	0.002	0.094	NA	NA	0.063	0.048	-0.001	0.09	NA	NA	0	0.047
-0.2	0.01	0.01	2	55-60	0.007	0.109	NA	NA	0.019	0.052	-0.001	0.101	NA	NA	0	0.05	0.002	0.1	NA	NA	0.017	0.048	0	0.099	NA	NA	0	0.047
-0.2	0.01	0.01	2	60-65	0.001	0.111	NA	NA	-0.011	0.052	-0.003	0.101	NA	NA	0	0.05	0.001	0.099	NA	NA	-0.014	0.048	0.001	0.099	NA	NA	0	0.047
-0.2	0.01	0.01	2	>65	0.005	0.102	NA	NA	-0.058	0.052	-0.002	0.097	NA	NA	0	0.05	0.002	0.088	NA	NA	-0.06	0.048	-0.005	0.087	NA	NA	0	0.047
-0.2	0.01	0.01	2	$TE_{age=60}$	0.004	0.051	0.004	0.056	0.004	0.052	-0.001	0.05	-0.001	0.054	0	0.05	0.002	0.048	0.002	0.051	0.002	0.048	-0.001	0.047	0	0.049	0	0.047
-0.2	0.01	0.01	2	vTE_{age+1}	0	0.007	-0.002	0.113	-0.006	0	0	0.006	0.004	0.107	0	0	0	0.006	-0.002	0.1	-0.006	0	0	0.006	0.002	0.092	0	0
-0.2	0.01	0.01	3	<55	-0.015	0.066	NA	NA	0.014	0.053	-0.004	0.227	NA	NA	0.001	0.05	-0.017	0.066	NA	NA	0.02	0.05	-0.017	0.155	NA	NA	-0.011	0.047
-0.2	0.01	0.01	3	55-60	0.003	0.121	NA	NA	-0.033	0.053	0.001	0.157	NA	NA	0.001	0.05	0	0.108	NA	NA	-0.027	0.05	-0.003	0.126	NA	NA	-0.011	0.047
-0.2	0.01	0.01	3	60-65	0.001	0.158	NA	NA	-0.063	0.053	-0.003	0.118	NA	NA	0.001	0.05	-0.009	0.129	NA	NA	-0.057	0.05	-0.003	0.102	NA	NA	-0.011	0.047
-0.2	0.01	0.01	3	>65	-0.009	0.214	NA	NA	-0.11	0.053	0.001	0.062	NA	NA	0.001	0.05	-0.01	0.143	NA	NA	-0.103	0.05	-0.004	0.062	NA	NA	-0.011	0.047
-0.2	0.01	0.01	3	$TE_{age=60}$	0.002	0.073	0.006	0.981	-0.048	0.053	-0.001	0.072	0.055	0.887	0.001	0.05	-0.003	0.059	-0.024	0.506	-0.042	0.05	-0.004	0.059	-0.02	0.504	-0.011	0.047
-0.2	0.01	0.01	3	vTE_{age+1}	0	0.007	0	0.122	-0.006	0	0	0.006	-0.007	0.11	0	0	0	0.005	-0.003	0.063	-0.006	0	0	0.005	0.002	0.063	0	0
-0.2	0.01	0.1	1	<55	0.004	0.105	NA	NA	0.002	0.06	0.004	0.104	NA	NA	0.004	0.06	0.002	0.094	NA	NA	0.002	0.054	0.004	0.089	NA	NA	0.003	0.051
-0.2	0.01	0.1	1	55-60	0	0.109	NA	NA	0.002	0.06	0.001	0.109	NA	NA	0.004	0.06	0.003	0.101	NA	NA	0.002	0.054	0.005	0.101	NA	NA	0.003	0.051
-0.2	0.01	0.1	1	60-65	-0.007	0.109	NA	NA	0.002	0.06	0.002	0.111	NA	NA	0.004	0.06	-0.001	0.097	NA	NA	0.002	0.054	-0.001	0.097	NA	NA	0.003	0.051
-0.2	0.01	0.1	1	>65	0.002	0.102	NA	NA	0.002	0.06	0.001	0.1	NA	NA	0.004	0.06	0.001	0.087	NA	NA	0.002	0.054	-0.001	0.088	NA	NA	0.003	0.051
-0.2	0.01	0.1	1	$TE_{age=60}$	0	0.059	0.003	0.064	0.002	0.06	0.002	0.06	0.003	0.065	0.004	0.06	0.001	0.054	0.002	0.056	0.002	0.054	0.002	0.051	0.002	0.054	0.003	0.051
-0.2	0.01	0.1	1	vTE_{age+1}	0	0.006	0	0.127	0	0	0	0.006	-0.002	0.129	0	0	0	0.006	-0.003	0.113	0	0	0	0.005	-0.002	0.116	0	0
-0.2	0.01	0.1	2	<55	0.009	0.108	NA	NA	0.069	0.061	0.004	0.109	NA	NA	0.002	0.06	-0.003	0.098	NA	NA	0.063	0.056	-0.002	0.092	NA	NA	0.001	0.055
-0.2	0.01	0.1	2	55-60	0.008	0.117	NA	NA	0.022	0.061	-0.006	0.113	NA	NA	0.002	0.06	0.004	0.104	NA	NA	0.017	0.056	0	0.099	NA	NA	0.001	0.055
-0.2	0.01	0.1	2	60-65	0.005	0.112	NA	NA	-0.008	0.061	0.003	0.114	NA	NA	0.002	0.06	0	0.098	NA	NA	-0.013	0.056	-0.002	0.097	NA	NA	0.001	0.055
-0.2	0.01	0.1	2	>65	0.004	0.105	NA	NA	-0.055	0.061	0	0.099	NA	NA	0.002	0.06	0.004	0.094	NA	NA	-0.06	0.056	0.001	0.097	NA	NA	0.001	0.055
-0.2	0.01	0.1	2	$TE_{age=60}$	0.006	0.061	0.006	0.065	0.007	0.061	0	0.06	0.002	0.063	0.002	0.06	0.001	0.056	0.001	0.058	0.002	0.056	-0.001	0.055	0.002	0.057	0.001	0.055
-0.2	0.01	0.1	2	vTE_{age+1}	0	0.006	-0.001	0.13	-0.006	0	0	0.007	0	0.127	0	0	0	0.006	0.002	0.116	-0.006	0	0	0.006	0.001	0.117	0	0
-0.2	0.01	0.1	3	<55	-0.016	0.073	NA	NA	0.014	0.06	-0.001	0.225	NA	NA	0.003	0.059	-0.01	0.07	NA	NA	0.028	0.054	-0.008	0.154	NA	NA	-0.006	0.054
-0.2	0.01	0.1	3	55-60	-0.005	0.122	NA	NA	-0.033	0.06	0.002	0.166	NA	NA	0.003	0.059	0.003	0.111	NA	NA	-0.019	0.054	0.001	0.135	NA	NA	-0.006	0.054
-0.2	0.01	0.1	3	60-65	0.002	0.161	NA	NA	-0.063	0.06	0.003	0.12	NA	NA	0.003	0.059	0.005	0.134	NA	NA	-0.049	0.054	0.001	0.115	NA	NA	-0.006	0.054
-0.2	0.01	0.1	3	>65	0.011	0.227	NA	NA	-0.11	0.06	0	0.069	NA	NA	0.003	0.059	-0.002	0.146	NA	NA	-0.096	0.054	0	0.067	NA	NA	-0.006	0.054
-0.2	0.01	0.1	3	$TE_{age=60}$	0.005	0.078	0.032	1.091	-0.048	0.06	0.003	0.079	-0.029	1.002	0.003	0.059	0.005	0.063	0.005	0.586	-0.034	0.054	0	0.067	0.005	0.584	-0.006	0.054
-0.2	0.01	0.1	3	vTE_{age+1}	0	0.007	0.004	0.136	-0.006	0	0	0.006	0.004	0.125	0	0	0	0.005	0	0.074	-0.006	0	0	0.006	0	0.073	0	0
-0.2	0.1	0.01	1	<55	-0.001	0.1	NA	NA	-0.001	0.052	-0.004	0.105	NA	NA	0	0.052	-0.005	0.088	NA	NA	-0.001	0.044	-0.008	0.094	NA	NA	0	0.045
-0.2	0.1	0.01	1	55-60	-0.004	0.106	NA	NA	-0.001	0.052	-0.003	0.107	NA	NA	0	0.052	0.002	0.093	NA	NA	-0.001	0.044	0.004	0.094	NA	NA	0	0.045
-0.2	0.1	0.01	1	60-65	-0.001	0.105	NA	NA	-0.001	0.052	-0.003	0.111	NA	NA	0	0.052	-0.003	0.094	NA	NA	-0.001	0.044	0.001	0.092	NA	NA	0	0.045
-0.2	0.1	0.01	1	>65	-0.002	0.098	NA	NA	-0.001	0.052	0.005	0.094	NA	NA	0	0.052	0.004	0.088	NA	NA	-0.001	0.044	0	0.087	NA	NA	0	0.045
-0.2	0.1	0.01	1	$TE_{age=60}$	-0.002	0.052	-0.002	0.057	-0.001	0.052	-0.001	0.052	0	0.054	0	0.052	-0.001	0.044	-0.001	0.047	-0.001	0.044	-0.001	0.045	0	0.047	0	0.045
-0.2	0.1	0.01	1	vTE_{age+1}	0	0.006	0	0.111	0	0	0	0.006	0.005	0.104	0	0	0	0.006	-0.003	0.094	0	0	0	0.006	0.003	0.09	0	0
-0.2	0.1	0.01	2	<55	0.007	0.103	NA	NA	0.066	0.054	0	0.103	NA	NA	0	0.052	0	0.093	NA	NA	0.061	0.049	0.003	0.091	NA	NA	0.003	0.047
-0.2	0.1	0.01	2	55-60	-0.001	0.111	NA	NA	0.02	0.054	-0.005	0.104	NA	NA	0	0.052	-0.005	0.1	NA	NA	0.014	0.049	0.004	0.097	NA	NA	0.003	0.047
-0.2	0.1	0.01	2	60-65	0.002	0.11	NA	NA	-0.01	0.054	0.001	0.108	NA	NA	0	0.052	-0.001	0.095	NA	NA	-0.016	0.049	0.001	0.094	NA	NA	0.003	0.047
-0.2	0.1	0.01	2	>65	0.011	0.1	NA	NA	-0.057	0.054	-0.001	0.097	NA	NA	0	0.052	0.003	0.089	NA	NA	-0.063	0.049	0.002	0.089	NA	NA	0.003	0.047
-0.2	0.1	0.01	2	$TE_{age=60}$	0.005	0.053	0.005	0.058	0.005	0.054	-0.001	0.052	0.001	0.056	0	0.052	-0.001	0.049	-0.002	0.052	-0.001	0.049	0.002	0.047	0.003	0.05	0.003	0.047
-0.2	0.1	0.01	2	vTE_{age+1}	0	0.007	0.006	0.109	-0.006	0	0	0.006	-0.002	0.108	0	0	0	0.006	0	0.098	-0.006	0	0	0.006	0.002	0.096	0	0

					Configuration 1												Configuration 2											
					A-C						B-C						A-C						B-C					
					IPD Poisson1		AD metareg		AD-netmeta		IPD Poisson1		AD metareg		AD-netmeta		IPD Poisson1		AD metareg		AD-netmeta		IPD Poisson1		AD metareg		AD-netmeta	
ttt	σ	τ	s	param	Bias	ESE	Bias	ESE	Bias	ESE	Bias	ESE	Bias	ESE	Bias	ESE	Bias	ESE	Bias	ESE	Bias	ESE	Bias	ESE	Bias	ESE	Bias	ESE
-0.2	0.1	0.01	3	<55	-0.017	0.068	NA	NA	0.011	0.052	0.001	0.229	NA	NA	-0.002	0.05	-0.016	0.065	NA	NA	0.022	0.048	-0.013	0.152	NA	NA	-0.008	0.045
-0.2	0.1	0.01	3	55-60	-0.002	0.114	NA	NA	-0.036	0.052	-0.004	0.158	NA	NA	-0.002	0.05	-0.001	0.109	NA	NA	-0.024	0.048	0.002	0.13	NA	NA	-0.008	0.045
-0.2	0.1	0.01	3	60-65	-0.005	0.163	NA	NA	-0.066	0.052	-0.005	0.113	NA	NA	-0.002	0.05	-0.001	0.131	NA	NA	-0.055	0.048	0	0.105	NA	NA	-0.008	0.045
-0.2	0.1	0.01	3	>65	-0.007	0.219	NA	NA	-0.113	0.052	-0.003	0.062	NA	NA	-0.002	0.05	-0.005	0.148	NA	NA	-0.101	0.048	0	0.06	NA	NA	-0.008	0.045
-0.2	0.1	0.01	3	$TE_{age=60}$	0.001	0.071	-0.007	0.916	-0.051	0.052	-0.003	0.072	0.01	0.866	-0.002	0.05	-0.001	0.059	-0.027	0.486	-0.04	0.048	0.001	0.057	-0.022	0.489	-0.008	0.045
-0.2	0.1	0.01	3	vTE_{age+1}	0	0.007	-0.001	0.114	-0.006	0	0	0.006	-0.001	0.108	0	0	0	0.006	-0.003	0.061	-0.006	0	0	0.005	0.003	0.061	0	0
-0.2	0.1	0.1	1	<55	0.007	0.108	NA	NA	0.005	0.061	-0.001	0.104	NA	NA	0.002	0.059	0.002	0.095	NA	NA	0.003	0.055	0.002	0.093	NA	NA	0.002	0.054
-0.2	0.1	0.1	1	55-60	0.008	0.112	NA	NA	0.005	0.061	0.008	0.105	NA	NA	0.002	0.059	-0.002	0.102	NA	NA	0.003	0.055	0.002	0.099	NA	NA	0.002	0.054
-0.2	0.1	0.1	1	60-65	0.002	0.105	NA	NA	0.005	0.061	-0.002	0.107	NA	NA	0.002	0.059	0.008	0.101	NA	NA	0.003	0.055	0.003	0.102	NA	NA	0.002	0.054
-0.2	0.1	0.1	1	>65	0.002	0.106	NA	NA	0.005	0.061	0.001	0.104	NA	NA	0.002	0.059	0	0.093	NA	NA	0.003	0.055	0.001	0.09	NA	NA	0.002	0.054
-0.2	0.1	0.1	1	$TE_{age=60}$	0.005	0.061	0.005	0.064	0.005	0.061	0.001	0.059	0.001	0.065	0.002	0.059	0.002	0.055	0.002	0.058	0.003	0.055	0.002	0.054	0.002	0.055	0.002	0.054
-0.2	0.1	0.1	1	vTE_{age+1}	0	0.006	0.002	0.115	0	0	0	0.006	0.005	0.13	0	0	0	0.006	-0.003	0.114	0	0	0	0.006	0	0.109	0	0
-0.2	0.1	0.1	2	<55	0.003	0.118	NA	NA	0.064	0.062	-0.003	0.106	NA	NA	0	0.059	0.005	0.098	NA	NA	0.064	0.056	-0.002	0.097	NA	NA	0.001	0.055
-0.2	0.1	0.1	2	55-60	-0.001	0.116	NA	NA	0.018	0.062	0.001	0.108	NA	NA	0	0.059	0.001	0.102	NA	NA	0.018	0.056	0.001	0.101	NA	NA	0.001	0.055
-0.2	0.1	0.1	2	60-65	0.008	0.111	NA	NA	-0.013	0.062	-0.002	0.11	NA	NA	0	0.059	0.002	0.103	NA	NA	-0.013	0.056	0.001	0.1	NA	NA	0.001	0.055
-0.2	0.1	0.1	2	>65	0	0.107	NA	NA	-0.059	0.062	-0.001	0.106	NA	NA	0	0.059	0.003	0.094	NA	NA	-0.059	0.056	0.002	0.094	NA	NA	0.001	0.055
-0.2	0.1	0.1	2	$TE_{age=60}$	0.003	0.062	0.002	0.066	0.002	0.062	-0.001	0.059	0	0.064	0	0.059	0.003	0.057	0.002	0.06	0.002	0.056	0	0.055	0.002	0.059	0.001	0.055
-0.2	0.1	0.1	2	vTE_{age+1}	0	0.007	0.006	0.128	-0.006	0	0	0.007	-0.001	0.135	0	0	0	0.006	-0.002	0.116	-0.006	0	0	0.006	0	0.12	0	0
-0.2	0.1	0.1	3	<55	-0.016	0.073	NA	NA	0.013	0.06	-0.004	0.238	NA	NA	0.002	0.06	-0.014	0.073	NA	NA	0.025	0.055	-0.012	0.16	NA	NA	-0.007	0.053
-0.2	0.1	0.1	3	55-60	0.003	0.126	NA	NA	-0.034	0.06	-0.002	0.161	NA	NA	0.002	0.06	0.002	0.11	NA	NA	-0.022	0.055	0.001	0.132	NA	NA	-0.007	0.053
-0.2	0.1	0.1	3	55-60	-0.002	0.16	NA	NA	-0.064	0.06	0.002	0.117	NA	NA	0.002	0.06	0.003	0.132	NA	NA	-0.052	0.055	0.002	0.11	NA	NA	-0.007	0.053
-0.2	0.1	0.1	3	>65	-0.01	0.229	NA	NA	-0.11	0.06	0.001	0.072	NA	NA	0.002	0.06	0.002	0.151	NA	NA	-0.099	0.055	0.001	0.067	NA	NA	-0.007	0.053
-0.2	0.1	0.1	3	$TE_{age=60}$	0.002	0.079	-0.004	1.051	-0.049	0.06	-0.001	0.079	-0.009	0.991	0.002	0.06	0.003	0.064	0.003	0.58	-0.037	0.055	0	0.064	-0.002	0.575	-0.007	0.053
-0.2	0.1	0.1	3	vTE_{age+1}	0	0.007	-0.001	0.131	-0.006	0	0	0.006	0.001	0.123	0	0	0	0.006	0	0.073	-0.006	0	0	0.005	0.001	0.072	0	0

Both configuration are 3 nodes (A-B-C) network, with no closed loop in configuration 1 (no A-B trials) and a closed loop in configuration 2.

IPD-Poisson is an Individual Patients' Data model based on Poisson's hierarchical model, AD-Metareg an aggregated-data based metaregression model an AD-Netmeta and aggregated-data model based on contrast

ttt : treatment effects as log(HR) with two possibilities : -0.2 and -0.5

σ: between-trial heterogeneity of baseline risk 0.01 or 0.1)

τ: between-trial heterogeneity of treatment effect 0.01 or 0.1)

S: scenario with three possibilities (1: no interaction, same age distribution; 2: interaction in AC, same age distribution; 3: interaction in AC, different age distribution)

Param: parameters estimated by the model with age in years as a 4-class categorical variable (<55, 55-60, 60-65, >65) or as a continuous variable ($TE_{age=60}$: marginal effect = log(HR) for a patient of age 60; vTE_{age+1}: the variation in log(HR) for a variation of one year of age)

NA: Not applicable
