# Supplementary material for: A frequentist one-step model for a simple network meta-analysis of time-to-event data in presence of an effect modifier
Source: PLoS One. 2021 Nov 1;16(11):e0259121. doi: 10.1371/journal.pone.0259121 (PMC8559936; doi:10.1371/journal.pone.0259121)
Supplement: S2 Table — Simulation results of the indirect A-B treatment effect estimation with age as a categorical variable (configuration 1) AD: aggregated values, IPD: individual patient’s data, ESE: empirical standard error, ASE: Average Standard Error, TE: treatment effect, σ between trial random effect for baseline risk, τ between-trial random effect for treatment effect. (PDF) [file pone.0259121.s004.pdf]

S2 table: Simulation results of the indirect A-B treatment effect estimation with age as a categorical variable (configuration 1)

						AD-Netmeta				IPD-Poisson1			
**TE**	**σ**	**τ**	**Scenario**	**Age (years)**	**True log(HR)**	**Mean**	**Bias**	**ESE**	**ASE**	**Mean**	**Bias**	**ESE**	**ASE**
**-0.5**	**0.01**	**0.01**	**1: None**	<55	0	0	0	0.076	0.08	0.004	0.004	0.143	0.151
				55-60	0	0	0	0.076	0.08	-0.008	-0.008	0.154	0.156
				60-65	0	0	0	0.076	0.08	0	0	0.154	0.156
				>65	0	0	0	0.076	0.08	0.001	0.001	0.143	0.155
			**2: Interaction**	<55	-0.154	0.006	0.16	0.081	0.085	-0.147	0.008	0.158	0.163
				55-60	-0.038	0.006	0.044	0.081	0.085	-0.037	0	0.162	0.166
				60-65	0.038	0.006	-0.032	0.081	0.085	0.044	0.006	0.159	0.166
				>65	0.154	0.006	-0.148	0.081	0.085	0.156	0.002	0.155	0.165
			**3: Both**	<55	-0.154	-0.12	0.034	0.084	0.086	-0.186	-0.031	0.259	0.255
				55-60	-0.038	-0.12	-0.083	0.084	0.086	-0.044	-0.006	0.214	0.259
				60-65	0.038	-0.12	-0.158	0.084	0.086	0.029	-0.009	0.213	0.26
				>65	0.154	-0.12	-0.275	0.084	0.086	0.121	-0.034	0.264	0.258
**-0.5**	**0.01**	**0.1**	**1: None**	<55	0	-0.002	-0.002	0.09	0.088	-0.002	-0.002	0.153	0.154
				55-60	0	-0.002	-0.002	0.09	0.088	0.001	0.001	0.157	0.159
				60-65	0	-0.002	-0.002	0.09	0.088	-0.007	-0.007	0.16	0.159
				>65	0	-0.002	-0.002	0.09	0.088	-0.001	-0.001	0.147	0.158
			**2: Interaction**	<55	-0.154	0.007	0.161	0.093	0.092	-0.142	0.012	0.169	0.166
				55-60	-0.038	0.007	0.045	0.093	0.092	-0.036	0.001	0.168	0.169
				60-65	0.038	0.007	-0.031	0.093	0.092	0.033	-0.005	0.171	0.169
				>65	0.154	0.007	-0.147	0.093	0.092	0.167	0.013	0.151	0.168
			**3: Both**	<55	-0.154	-0.122	0.033	0.094	0.094	-0.186	-0.031	0.268	0.257
				55-60	-0.038	-0.122	-0.084	0.094	0.094	-0.044	-0.007	0.214	0.262
				60-65	0.038	-0.122	-0.159	0.094	0.094	0.037	-0.001	0.215	0.262
				>65	0.154	-0.122	-0.276	0.094	0.094	0.124	-0.031	0.269	0.261
**-0.5**	**0.1**	**0.01**	**1: None**	<55	0	0.004	0.004	0.077	0.08	-0.002	-0.002	0.149	0.152
				55-60	0	0.004	0.004	0.077	0.08	0.003	0.003	0.156	0.157
				60-65	0	0.004	0.004	0.077	0.08	0.015	0.015	0.16	0.156
				>65	0	0.004	0.004	0.077	0.08	0.002	0.002	0.137	0.156
			**2: Interaction**	<55	-0.154	0.002	0.156	0.078	0.086	-0.156	-0.002	0.162	0.164
				55-60	-0.038	0.002	0.04	0.078	0.086	-0.034	0.004	0.164	0.167
				60-65	0.038	0.002	-0.036	0.078	0.086	0.035	-0.003	0.162	0.167
				>65	0.154	0.002	-0.152	0.078	0.086	0.154	0	0.151	0.166
			**3: Both**	<55	-0.154	-0.124	0.031	0.081	0.086	-0.18	-0.026	0.257	0.256
				55-60	-0.038	-0.124	-0.086	0.081	0.086	-0.047	-0.009	0.212	0.26
				60-65	0.038	-0.124	-0.162	0.081	0.086	0.019	-0.019	0.229	0.26
				>65	0.154	-0.124	-0.278	0.081	0.086	0.121	-0.034	0.268	0.259
**-0.5**	**0.1**	**0.1**	**1: None**	<55	0	0.002	0.002	0.086	0.088	0.013	0.013	0.151	0.155
				55-60	0	0.002	0.002	0.086	0.088	0	0	0.167	0.16
				60-65	0	0.002	0.002	0.086	0.088	-0.003	-0.003	0.159	0.16
				>65	0	0.002	0.002	0.086	0.088	-0.001	-0.001	0.147	0.159
			**2: Interaction**	<55	-0.154	0.001	0.155	0.096	0.093	-0.15	0.004	0.168	0.167
				55-60	-0.038	0.001	0.039	0.096	0.093	-0.039	-0.002	0.174	0.17

						AD-Netmeta				IPD-Poisson1			
**TE**	**σ**	**τ**	**Scenario**	**Age (years)**	**True log(HR)**	**Mean**	**Bias**	**ESE**	**ASE**	**Mean**	**Bias**	**ESE**	**ASE**
				60-65	0.038	0.001	-0.037	0.096	0.093	0.034	-0.004	0.171	0.17
				>65	0.154	0.001	-0.153	0.096	0.093	0.154	0	0.162	0.169
			**3: Both**	<55	-0.154	-0.119	0.035	0.09	0.095	-0.197	-0.043	0.253	0.258
				55-60	-0.038	-0.119	-0.081	0.09	0.095	-0.039	-0.002	0.216	0.262
				60-65	0.038	-0.119	-0.157	0.09	0.095	0.03	-0.008	0.227	0.263
				>65	0.154	-0.119	-0.274	0.09	0.095	0.149	-0.005	0.26	0.262
**-0.2**	**0.01**	**0.01**	**1: None**	<55	0	-0.002	-0.002	0.073	0.076	0.006	0.006	0.138	0.145
				55-60	0	-0.002	-0.002	0.073	0.076	-0.005	-0.005	0.143	0.152
				60-65	0	-0.002	-0.002	0.073	0.076	-0.005	-0.005	0.151	0.152
				>65	0	-0.002	-0.002	0.073	0.076	-0.006	-0.006	0.134	0.151
			**2: Interaction**	<55	-0.062	0.004	0.066	0.073	0.078	-0.06	0.002	0.144	0.148
				55-60	-0.015	0.004	0.019	0.073	0.078	-0.007	0.008	0.149	0.155
				60-65	0.015	0.004	-0.011	0.073	0.078	0.019	0.004	0.152	0.155
				>65	0.062	0.004	-0.058	0.073	0.078	0.068	0.006	0.142	0.154
			**3: Both**	<55	-0.062	-0.049	0.013	0.073	0.079	-0.073	-0.011	0.237	0.245
				55-60	-0.015	-0.049	-0.034	0.073	0.079	-0.014	0.001	0.201	0.251
				60-65	0.015	-0.049	-0.064	0.073	0.079	0.019	0.003	0.197	0.252
				>65	0.062	-0.049	-0.11	0.073	0.079	0.052	-0.01	0.224	0.251
**-0.2**	**0.01**	**0.1**	**1: None**	<55	0	-0.002	-0.002	0.084	0.085	0	0	0.149	0.148
				55-60	0	-0.002	-0.002	0.084	0.085	-0.001	-0.001	0.153	0.156
				60-65	0	-0.002	-0.002	0.084	0.085	-0.01	-0.01	0.151	0.156
				>65	0	-0.002	-0.002	0.084	0.085	0.001	0.001	0.143	0.155
			**2: Interaction**	<55	-0.062	0.005	0.066	0.087	0.087	-0.057	0.004	0.154	0.152
				55-60	-0.015	0.005	0.02	0.087	0.087	-0.001	0.014	0.164	0.159
				60-65	0.015	0.005	-0.011	0.087	0.087	0.016	0.001	0.156	0.159
				>65	0.062	0.005	-0.057	0.087	0.087	0.067	0.005	0.146	0.158
			**3: Both**	<55	-0.062	-0.051	0.011	0.084	0.087	-0.077	-0.015	0.235	0.247
				55-60	-0.015	-0.051	-0.036	0.084	0.087	-0.022	-0.007	0.202	0.253
				60-65	0.015	-0.051	-0.066	0.084	0.087	0.014	-0.001	0.202	0.254
				>65	0.062	-0.051	-0.113	0.084	0.087	0.072	0.01	0.239	0.253
**-0.2**	**0.1**	**0.01**	**1: None**	<55	0	-0.001	-0.001	0.074	0.076	0.003	0.003	0.15	0.145
				55-60	0	-0.001	-0.001	0.074	0.076	-0.002	-0.002	0.15	0.152
				60-65	0	-0.001	-0.001	0.074	0.076	0.001	0.001	0.151	0.152
				>65	0	-0.001	-0.001	0.074	0.076	-0.007	-0.007	0.133	0.152
			**2: Interaction**	<55	-0.062	0.005	0.067	0.076	0.078	-0.055	0.007	0.144	0.149
				55-60	-0.015	0.005	0.02	0.076	0.078	-0.011	0.004	0.148	0.156
				60-65	0.015	0.005	-0.01	0.076	0.078	0.016	0.001	0.152	0.156
				>65	0.062	0.005	-0.057	0.076	0.078	0.074	0.012	0.142	0.155
			**3: Both**	<55	-0.062	-0.049	0.013	0.074	0.078	-0.08	-0.018	0.239	0.245
				55-60	-0.015	-0.049	-0.034	0.074	0.078	-0.014	0.001	0.197	0.251
				60-65	0.015	-0.049	-0.064	0.074	0.078	0.015	0	0.2	0.252
				>65	0.062	-0.049	-0.111	0.074	0.078	0.057	-0.004	0.227	0.251
**-0.2**	**0.1**	**0.1**	**1: None**	<55	0	0.004	0.004	0.085	0.085	0.008	0.008	0.148	0.149
				55-60	0	0.004	0.004	0.085	0.085	0	0	0.15	0.156

TE	σ	τ	Scenario	Age (years)	True log(HR)	AD-Netmeta Mean	AD-Netmeta Bias	AD-Netmeta ESE	AD-Netmeta ASE	IPD-Poisson1 Mean	IPD-Poisson1 Bias	IPD-Poisson1 ESE	IPD-Poisson1 ASE
				60-65	0	0.004	0.004	0.085	0.085	0.004	0.004	0.148	0.156
				>65	0	0.004	0.004	0.085	0.085	0.001	0.001	0.148	0.156
			**2: Interaction**	<55	-0.062	0.003	0.064	0.086	0.087	-0.055	0.007	0.158	0.153
				55-60	-0.015	0.003	0.018	0.086	0.087	-0.016	-0.001	0.16	0.16
				60-65	0.015	0.003	-0.013	0.086	0.087	0.025	0.009	0.157	0.159
				>65	0.062	0.003	-0.059	0.086	0.087	0.062	0	0.149	0.159
			**3: Both**	<55	-0.062	-0.05	0.011	0.087	0.087	-0.074	-0.012	0.251	0.248
				55-60	-0.015	-0.05	-0.035	0.087	0.087	-0.01	0.005	0.205	0.253
				60-65	0.015	-0.05	-0.065	0.087	0.087	0.011	-0.004	0.197	0.254
				>65	0.062	-0.05	-0.112	0.087	0.087	0.051	-0.01	0.242	0.253

AD: aggregated values, IPD: individual patient's data, ESE: empirical standard error, ASE: Average Standard Error, TE: treatment effect, σ between trial random effect for baseline risk, τ between-trial random effect for treatment effect
