# Supplementary material for: A frequentist one-step model for a simple network meta-analysis of time-to-event data in presence of an effect modifier
Source: PLoS One. 2021 Nov 1;16(11):e0259121. doi: 10.1371/journal.pone.0259121 (PMC8559936; doi:10.1371/journal.pone.0259121)
Supplement: S3 Table — Simulation results of the mixed (direct/indirect) A-B treatment effect estimation with age as a categorical variable (configuration 2) AD: aggregated values, IPD: individual patient’s data, ESE: empirical standard error, ASE: Average Standard Error, TE: treatment effect, σ between trial random effect for baseline risk, τ between-trial random effect for treatment effect. (PDF) [file pone.0259121.s005.pdf]

S3 table: Simulation results of the mixed (direct/indirect) A-B treatment effect estimation with age as a categorical variable (configuration 2)

						AD-Netmeta				IPD-Poisson1			
**TE**	**σ**	**τ**	**Scenario**	**Age (years)**	**True log(HR)**	**Mean**	**Bias**	**ESE**	**ASE**	**Mean**	**Bias**	**ESE**	**ASE**
**-0.5**	**0.01**	**0.01**	**1: None**	<55	0	-0.003	-0.003	0.057	0.061	-0.005	-0.005	0.112	0.15
				55-60	0	-0.003	-0.003	0.057	0.061	-0.003	-0.003	0.125	0.154
				60-65	0	-0.003	-0.003	0.057	0.061	-0.002	-0.002	0.115	0.154
				>65	0	-0.003	-0.003	0.057	0.061	-0.002	-0.002	0.105	0.154
			**2: Interaction**	<55	-0.154	0.003	0.157	0.064	0.067	-0.153	0.002	0.129	0.162
				55-60	-0.038	0.003	0.04	0.064	0.067	-0.033	0.005	0.135	0.165
				60-65	0.038	0.003	-0.035	0.064	0.067	0.036	-0.002	0.131	0.165
				>65	0.154	0.003	-0.152	0.064	0.067	0.157	0.003	0.113	0.164
			**3: Both**	<55	-0.154	-0.075	0.079	0.065	0.067	-0.164	-0.01	0.156	0.199
				55-60	-0.038	-0.075	-0.037	0.065	0.067	-0.049	-0.011	0.153	0.204
				60-65	0.038	-0.075	-0.113	0.065	0.067	0.033	-0.004	0.152	0.205
				>65	0.154	-0.075	-0.229	0.065	0.067	0.144	-0.01	0.16	0.204
**-0.5**	**0.01**	**0.1**	**1: None**	<55	0	-0.001	-0.001	0.066	0.068	0.001	0.001	0.121	0.153
				55-60	0	-0.001	-0.001	0.066	0.068	-0.002	-0.002	0.128	0.157
				60-65	0	-0.001	-0.001	0.066	0.068	-0.007	-0.007	0.121	0.157
				>65	0	-0.001	-0.001	0.066	0.068	0.003	0.003	0.113	0.157
			**2: Interaction**	<55	-0.154	0.005	0.16	0.072	0.073	-0.151	0.003	0.131	0.165
				55-60	-0.038	0.005	0.043	0.072	0.073	-0.029	0.009	0.138	0.168
				60-65	0.038	0.005	-0.032	0.072	0.073	0.041	0.003	0.133	0.168
				>65	0.154	0.005	-0.149	0.072	0.073	0.159	0.004	0.12	0.167
			**3: Both**	<55	-0.154	-0.072	0.082	0.074	0.074	-0.165	-0.011	0.162	0.203
				55-60	-0.038	-0.072	-0.034	0.074	0.074	-0.044	-0.007	0.156	0.207
				60-65	0.038	-0.072	-0.11	0.074	0.074	0.027	-0.011	0.153	0.208
				>65	0.154	-0.072	-0.226	0.074	0.074	0.154	0	0.153	0.207
**-0.5**	**0.1**	**0.01**	**1: None**	<55	0	0.001	0.001	0.058	0.062	0.003	0.003	0.113	0.15
				55-60	0	0.001	0.001	0.058	0.062	0.004	0.004	0.118	0.155
				60-65	0	0.001	0.001	0.058	0.062	-0.001	-0.001	0.114	0.155
				>65	0	0.001	0.001	0.058	0.062	-0.001	-0.001	0.11	0.154
			**2: Interaction**	<55	-0.154	0.001	0.155	0.063	0.067	-0.154	0	0.125	0.162
				55-60	-0.038	0.001	0.039	0.063	0.067	-0.045	-0.007	0.13	0.165
				60-65	0.038	0.001	-0.037	0.063	0.067	0.04	0.002	0.128	0.165
				>65	0.154	0.001	-0.153	0.063	0.067	0.16	0.006	0.119	0.164
			**3: Both**	<55	-0.154	-0.073	0.081	0.064	0.067	-0.172	-0.018	0.162	0.2
				55-60	-0.038	-0.073	-0.035	0.064	0.067	-0.04	-0.002	0.148	0.205
				60-65	0.038	-0.073	-0.111	0.064	0.067	0.035	-0.003	0.149	0.205
				>65	0.154	-0.073	-0.227	0.064	0.067	0.148	-0.006	0.15	0.204
**-0.5**	**0.1**	**0.1**	**1: None**	<55	0	0	0	0.068	0.068	0.004	0.004	0.122	0.153
				55-60	0	0	0	0.068	0.068	-0.001	-0.001	0.128	0.158
				60-65	0	0	0	0.068	0.068	0	0	0.125	0.158
				>65	0	0	0	0.068	0.068	-0.005	-0.005	0.115	0.157
			**2: Interaction**	<55	-0.154	0.004	0.159	0.074	0.073	-0.153	0.001	0.131	0.165
				55-60	-0.038	0.004	0.042	0.074	0.073	-0.037	0.001	0.135	0.168

TE	σ	τ	Scenario	Age (years)	True log(HR)	AD-Netmeta Mean	Bias	ESE	ASE	IPD-Poisson1 Mean	Bias	ESE	ASE
				60-65	0.038	0.004	-0.033	0.074	0.073	0.049	0.011	0.143	0.168
				>65	0.154	0.004	-0.15	0.074	0.073	0.156	0.001	0.122	0.167
			**3: Both**	<55	-0.154	-0.073	0.081	0.071	0.075	-0.169	-0.014	0.159	0.204
				55-60	-0.038	-0.073	-0.035	0.071	0.075	-0.034	0.004	0.155	0.209
				60-65	0.038	-0.073	-0.111	0.071	0.075	0.036	-0.002	0.148	0.209
				>65	0.154	-0.073	-0.227	0.071	0.075	0.147	-0.007	0.154	0.208
**-0.2**	**0.01**	**0.01**	**1: None**	<55	0	-0.001	-0.001	0.059	0.06	0.004	0.004	0.115	0.144
				55-60	0	-0.001	-0.001	0.059	0.06	-0.007	-0.007	0.117	0.15
				60-65	0	-0.001	-0.001	0.059	0.06	-0.002	-0.002	0.118	0.15
				>65	0	-0.001	-0.001	0.059	0.06	-0.001	-0.001	0.112	0.149
			**2: Interaction**	<55	-0.062	0.002	0.064	0.059	0.061	-0.059	0.003	0.113	0.147
				55-60	-0.015	0.002	0.017	0.059	0.061	-0.013	0.002	0.118	0.154
				60-65	0.015	0.002	-0.013	0.059	0.061	0.015	0	0.124	0.154
				>65	0.062	0.002	-0.06	0.059	0.061	0.069	0.007	0.108	0.153
			**3: Both**	<55	-0.062	-0.03	0.031	0.06	0.062	-0.062	0	0.152	0.184
				55-60	-0.015	-0.03	-0.015	0.06	0.062	-0.012	0.003	0.137	0.191
				60-65	0.015	-0.03	-0.045	0.06	0.062	0.009	-0.006	0.139	0.192
				>65	0.062	-0.03	-0.092	0.06	0.062	0.056	-0.006	0.135	0.191
**-0.2**	**0.01**	**0.1**	**1: None**	<55	0	-0.001	-0.001	0.066	0.067	-0.002	-0.002	0.116	0.147
				55-60	0	-0.001	-0.001	0.066	0.067	-0.002	-0.002	0.125	0.153
				60-65	0	-0.001	-0.001	0.066	0.067	0.001	0.001	0.12	0.153
				>65	0	-0.001	-0.001	0.066	0.067	0.003	0.003	0.113	0.153
			**2: Interaction**	<55	-0.062	0	0.062	0.067	0.068	-0.063	-0.001	0.114	0.151
				55-60	-0.015	0	0.015	0.067	0.068	-0.011	0.004	0.126	0.157
				60-65	0.015	0	-0.015	0.067	0.068	0.017	0.002	0.123	0.157
				>65	0.062	0	-0.061	0.067	0.068	0.065	0.003	0.117	0.156
			**3: Both**	<55	-0.062	-0.028	0.034	0.066	0.068	-0.063	-0.002	0.155	0.188
				55-60	-0.015	-0.028	-0.013	0.066	0.068	-0.014	0.001	0.147	0.195
				60-65	0.015	-0.028	-0.043	0.066	0.068	0.019	0.004	0.139	0.195
				>65	0.062	-0.028	-0.09	0.066	0.068	0.059	-0.003	0.147	0.194
**-0.2**	**0.1**	**0.01**	**1: None**	<55	0	0	0	0.056	0.06	0.003	0.003	0.11	0.144
				55-60	0	0	0	0.056	0.06	-0.002	-0.002	0.116	0.151
				60-65	0	0	0	0.056	0.06	-0.005	-0.005	0.117	0.15
				>65	0	0	0	0.056	0.06	0.004	0.004	0.108	0.15
			**2: Interaction**	<55	-0.062	-0.004	0.058	0.059	0.061	-0.065	-0.004	0.114	0.148
				55-60	-0.015	-0.004	0.011	0.059	0.061	-0.023	-0.008	0.122	0.154
				60-65	0.015	-0.004	-0.019	0.059	0.061	0.013	-0.002	0.118	0.154
				>65	0.062	-0.004	-0.066	0.059	0.061	0.063	0.001	0.109	0.153
			**3: Both**	<55	-0.062	-0.032	0.03	0.057	0.061	-0.064	-0.003	0.15	0.184
				55-60	-0.015	-0.032	-0.017	0.057	0.061	-0.018	-0.003	0.142	0.191
				60-65	0.015	-0.032	-0.047	0.057	0.061	0.014	-0.001	0.134	0.191
				>65	0.062	-0.032	-0.094	0.057	0.061	0.057	-0.005	0.145	0.19
**-0.2**	**0.1**	**0.1**	**1: None**	<55	0	0	0	0.068	0.067	0.001	0.001	0.117	0.148
				55-60	0	0	0	0.068	0.067	-0.004	-0.004	0.122	0.154

TE	σ	τ	Scenario	Age (years)	True log(HR)	AD-Netmeta Mean	Bias	ESE	ASE	IPD-Poisson1 Mean	Bias	ESE	ASE
				60-65	0	0	0	0.068	0.067	0.005	0.005	0.12	0.154
				>65	0	0	0	0.068	0.067	0	0	0.115	0.154
			**2: Interaction**	<55	-0.062	0.001	0.063	0.068	0.069	-0.055	0.007	0.121	0.151
				55-60	-0.015	0.001	0.016	0.068	0.069	-0.016	0	0.129	0.158
				60-65	0.015	0.001	-0.014	0.068	0.069	0.016	0.001	0.119	0.157
				>65	0.062	0.001	-0.06	0.068	0.069	0.063	0.001	0.118	0.157
			**3: Both**	<55	-0.062	-0.03	0.032	0.068	0.068	-0.064	-0.002	0.154	0.188
				55-60	-0.015	-0.03	-0.015	0.068	0.068	-0.014	0.001	0.145	0.195
				60-65	0.015	-0.03	-0.045	0.068	0.068	0.016	0.001	0.146	0.196
				>65	0.062	-0.03	-0.091	0.068	0.068	0.063	0.001	0.149	0.195

AD: aggregated values, IPD: individual patient's data, ESE: empirical standard error, ASE: Average Standard Error, TE: treatment effect, σ between trial random effect for baseline risk, τ between-trial random effect for treatment effect
