# Supplementary material for: A frequentist one-step model for a simple network meta-analysis of time-to-event data in presence of an effect modifier
Source: PLoS One. 2021 Nov 1;16(11):e0259121. doi: 10.1371/journal.pone.0259121 (PMC8559936; doi:10.1371/journal.pone.0259121)
Supplement: S4 Table — Simulation results of the indirect A-B treatment effect estimation (configuration 1) and mixed (direct/indirect) estimation (configuration 2) with age as a continuous variable Configuration 1 represents a 3 arms network with B-C and A-C trials from which we estimate the indirect treatment effect AB, configuration 2 represent a 3 arms network with A-B, A-C and B-C trials from which we estimate the mixed (direct and indirect) treatment effect AB. AD: aggregated values, IPD: individual patient’s data, ESE: empirical standard error, ASE: Average Standard Error, TE: treatment effect, σ between trial random effect for baseline risk, τ between-trial random effect for treatment effect, TEage = 60: marginal effect = log(HR) for a patient of age 60; vTEage+1: the variation in log(HR) for a variation of one year of age). (PDF) [file pone.0259121.s006.pdf]

S4 Table: Simulation results of the indirect A-B treatment effect estimation (configuration 1) and mixed (direct/indirect) estimation (configuration 2) with age as a continuous variable

						IPD-Poisson1				AD-Metareg				AD-Netmeta			
TE	σ	τ	Scenario	Treatment effect	True log(HR)	Mean	Bias	ESE	ASE	Mean	Bias	ESE	ASE	Mean	Bias	ESE	ASE
**Configuration 1**																	
-0.5	0.01	0.01	**1: None**	$TE_{age=60}$	0	0	0	0.075	0.006	0.001	0.001	0.081	0.007	0	0	0.076	0.006
				vTE_{age+1}	0	0	0	0.009	0	0.009	0.009	0.159	0.025	0	0	0	0
			**2: Interaction**	$TE_{age=60}$	0	0.004	0.004	0.08	0.006	0.005	0.005	0.085	0.007	0.006	0.006	0.081	0.007
				vTE_{age+1}	0.016	0.015	0	0.01	0	0.019	0.004	0.17	0.029	0	-0.016	0	0
			**3: Both**	$TE_{age=60}$	0	-0.003	-0.003	0.113	0.013	-0.041	-0.041	1.381	1.907	-0.12	-0.12	0.084	0.022
				vTE_{age+1}	0.016	0.015	0	0.01	0	0.013	-0.002	0.179	0.032	0	-0.016	0	0
-0.5	0.01	0.1	**1: None**	$TE_{age=60}$	0	-0.002	-0.002	0.087	0.008	0	0	0.096	0.009	-0.002	-0.002	0.09	0.008
				vTE_{age+1}	0	0	0	0.009	0	-0.008	-0.008	0.182	0.033	0	0	0	0
			**2: Interaction**	$TE_{age=60}$	0	0.005	0.005	0.092	0.009	0.007	0.007	0.098	0.01	0.007	0.007	0.093	0.009
				vTE_{age+1}	0.016	0.016	0	0.01	0	0.014	-0.002	0.195	0.038	0	-0.016	0	0
			**3: Both**	$TE_{age=60}$	0	0.002	0.002	0.12	0.014	0.029	0.029	1.6	2.559	-0.122	-0.122	0.094	0.024
				vTE_{age+1}	0.016	0.015	-0.001	0.01	0	0.01	-0.006	0.199	0.04	0	-0.016	0	0
-0.5	0.1	0.01	**1: None**	$TE_{age=60}$	0	0.004	0.004	0.077	0.006	0.005	0.005	0.081	0.007	0.004	0.004	0.077	0.006
				vTE_{age+1}	0	0	0	0.009	0	-0.007	-0.007	0.158	0.025	0	0	0	0
			**2: Interaction**	$TE_{age=60}$	0	-0.001	-0.001	0.077	0.006	0	0	0.084	0.007	0.002	0.002	0.078	0.006
				vTE_{age+1}	0.016	0.015	0	0.01	0	0.01	-0.005	0.178	0.032	0	-0.016	0	0
			**3: Both**	$TE_{age=60}$	0	-0.004	-0.004	0.112	0.012	0.005	0.005	1.38	1.903	-0.124	-0.124	0.081	0.022
				vTE_{age+1}	0.016	0.015	0	0.01	0	0.015	-0.001	0.175	0.03	0	-0.016	0	0
-0.5	0.1	0.1	**1: None**	$TE_{age=60}$	0	0.003	0.003	0.085	0.007	0.005	0.005	0.092	0.008	0.002	0.002	0.086	0.007
				vTE_{age+1}	0	0	0	0.009	0	0.003	0.003	0.183	0.033	0	0	0	0
			**2: Interaction**	$TE_{age=60}$	0	-0.001	-0.001	0.096	0.009	-0.001	-0.001	0.102	0.01	0.001	0.001	0.096	0.009
				vTE_{age+1}	0.016	0.015	0	0.01	0	0.02	0.005	0.196	0.038	0	-0.016	0	0
			**3: Both**	$TE_{age=60}$	0	0.005	0.005	0.117	0.014	0.06	0.06	1.582	2.504	-0.119	-0.119	0.09	0.022
				vTE_{age+1}	0.016	0.016	0.001	0.01	0	0.012	-0.004	0.195	0.038	0	-0.016	0	0
-0.2	0.01	0.01	**1: None**	$TE_{age=60}$	0	-0.003	-0.003	0.072	0.005	-0.003	-0.003	0.078	0.006	-0.002	-0.002	0.073	0.005
				vTE_{age+1}	0	0	0	0.009	0	0.002	0.002	0.15	0.023	0	0	0	0
			**2: Interaction**	$TE_{age=60}$	0	0.005	0.005	0.071	0.005	0.005	0.005	0.079	0.006	0.004	0.004	0.073	0.005

TE	σ	τ	Scenario	Treatment effect	True log(HR)	IPD-Poisson1 Mean	Bias	ESE	ASE	AD-Metareg Mean	Bias	ESE	ASE	AD-Netmeta Mean	Bias	ESE	ASE
				vTE_{age+1}	0.006	0.006	0	0.009	0	0	-0.006	0.154	0.024	0	-0.006	0	0
			**3: Both**	$TE_{age=60}$	0	0.004	0.004	0.102	0.01	-0.049	-0.049	1.311	1.72	-0.049	-0.049	0.073	0.008
				vTE_{age+1}	0.006	0.006	0	0.009	0	0.013	0.007	0.166	0.028	0	-0.006	0	0
-0.2	0.01	0.1	**1: None**	$TE_{age=60}$	0	-0.002	-0.002	0.083	0.007	-0.001	-0.001	0.091	0.008	-0.002	-0.002	0.084	0.007
				vTE_{age+1}	0	0	0	0.009	0	0.002	0.002	0.181	0.033	0	0	0	0
			**2: Interaction**	$TE_{age=60}$	0	0.006	0.006	0.087	0.008	0.004	0.004	0.092	0.009	0.005	0.005	0.087	0.008
				vTE_{age+1}	0.006	0.006	0	0.009	0	0.006	-0.001	0.178	0.032	0	-0.006	0	0
			**3: Both**	$TE_{age=60}$	0	0.002	0.002	0.109	0.012	0.062	0.062	1.498	2.246	-0.051	-0.051	0.084	0.01
				vTE_{age+1}	0.006	0.007	0.001	0.009	0	0.006	0	0.182	0.033	0	-0.006	0	0
-0.2	0.1	0.01	**1: None**	$TE_{age=60}$	0	-0.001	-0.001	0.074	0.005	-0.002	-0.002	0.079	0.006	-0.001	-0.001	0.074	0.005
				vTE_{age+1}	0	0	0	0.009	0	-0.005	-0.005	0.155	0.024	0	0	0	0
			**2: Interaction**	$TE_{age=60}$	0	0.006	0.006	0.076	0.006	0.004	0.004	0.082	0.007	0.005	0.005	0.076	0.006
				vTE_{age+1}	0.006	0.006	0	0.009	0	0.014	0.008	0.153	0.024	0	-0.006	0	0
			**3: Both**	$TE_{age=60}$	0	0.004	0.004	0.103	0.011	-0.017	-0.017	1.255	1.573	-0.049	-0.049	0.074	0.008
				vTE_{age+1}	0.006	0.006	0	0.009	0	0.007	0.001	0.158	0.025	0	-0.006	0	0
-0.2	0.1	0.1	**1: None**	$TE_{age=60}$	0	0.003	0.003	0.085	0.007	0.004	0.004	0.092	0.008	0.004	0.004	0.085	0.007
				vTE_{age+1}	0	0	0	0.009	0	-0.002	-0.002	0.17	0.029	0	0	0	0
			**2: Interaction**	$TE_{age=60}$	0	0.004	0.004	0.086	0.007	0.002	0.002	0.093	0.009	0.003	0.003	0.086	0.007
				vTE_{age+1}	0.006	0.006	0	0.01	0	0.013	0.006	0.187	0.035	0	-0.006	0	0
			**3: Both**	$TE_{age=60}$	0	0.003	0.003	0.114	0.013	0.005	0.005	1.427	2.033	-0.05	-0.05	0.087	0.01
				vTE_{age+1}	0.006	0.006	0	0.009	0	0.004	-0.002	0.182	0.033	0	-0.006	0	0
**Configuration 2**																	
-0.5	0.01	0.01	**1: None**	$TE_{age=60}$	0	-0.003	-0.003	0.057	0.003	-0.002	-0.002	0.06	0.004	-0.003	-0.003	0.057	0.003
				vTE_{age+1}	0	0	0	0.007	0	-0.001	-0.001	0.12	0.014	0	0	0	0
			**2: Interaction**	$TE_{age=60}$	0	0.002	0.002	0.064	0.004	0.003	0.003	0.068	0.005	0.003	0.003	0.064	0.004
				vTE_{age+1}	0.016	0.016	0	0.008	0	0.013	-0.003	0.129	0.017	0	-0.016	0	0
			**3: Both**	$TE_{age=60}$	0	-0.001	-0.001	0.078	0.006	0.001	0.001	0.11	0.012	-0.075	-0.075	0.065	0.01
				vTE_{age+1}	0.016	0.016	0	0.008	0	0.025	0.009	0.135	0.018	0	-0.016	0	0
-0.5	0.01	0.1	**1: None**	$TE_{age=60}$	0	-0.001	-0.001	0.067	0.004	0	0	0.069	0.005	-0.001	-0.001	0.066	0.004
				vTE_{age+1}	0	0	0	0.007	0	0.005	0.005	0.141	0.02	0	0	0	0
			**2: Interaction**	$TE_{age=60}$	0	0.004	0.004	0.072	0.005	0.005	0.005	0.078	0.006	0.005	0.005	0.072	0.005
				vTE_{age+1}	0.016	0.016	0	0.008	0	0.017	0.001	0.148	0.022	0	-0.016	0	0

						IPD-Poisson1				AD-Metareg				AD-Netmeta			
TE	σ	τ	Scenario	Treatment effect	True log(HR)	Mean	Bias	ESE	ASE	Mean	Bias	ESE	ASE	Mean	Bias	ESE	ASE
			**3: Both**	$TE_{age=60}$	0	0.001	0.001	0.084	0.007	-0.001	-0.001	0.128	0.016	-0.072	-0.072	0.074	0.011
				vTE_{age+1}	0.016	0.016	0	0.008	0	0.01	-0.005	0.151	0.023	0	-0.016	0	0
-0.5	0.1	0.01	**1: None**	$TE_{age=60}$	0	0.001	0.001	0.057	0.003	0	0	0.061	0.004	0.001	0.001	0.058	0.003
				vTE_{age+1}	0	0	0	0.007	0	0.004	0.004	0.127	0.016	0	0	0	0
			**2: Interaction**	$TE_{age=60}$	0	0	0	0.064	0.004	0	0	0.069	0.005	0.001	0.001	0.063	0.004
				vTE_{age+1}	0.016	0.016	0	0.008	0	0.011	-0.005	0.134	0.018	0	-0.016	0	0
			**3: Both**	$TE_{age=60}$	0	0.002	0.002	0.076	0.006	0	0	0.11	0.012	-0.073	-0.073	0.064	0.009
				vTE_{age+1}	0.016	0.016	0	0.008	0	0.026	0.011	0.129	0.017	0	-0.016	0	0
-0.5	0.1	0.1	**1: None**	$TE_{age=60}$	0	-0.001	-0.001	0.068	0.005	0	0	0.073	0.005	0	0	0.068	0.005
				vTE_{age+1}	0	0	0	0.007	0	-0.001	-0.001	0.136	0.019	0	0	0	0
			**2: Interaction**	$TE_{age=60}$	0	0.003	0.003	0.074	0.005	0.004	0.004	0.078	0.006	0.004	0.004	0.074	0.005
				vTE_{age+1}	0.016	0.016	0	0.008	0	0.013	-0.002	0.15	0.023	0	-0.016	0	0
			**3: Both**	$TE_{age=60}$	0	0.005	0.005	0.084	0.007	0.005	0.005	0.128	0.016	-0.073	-0.073	0.071	0.01
				vTE_{age+1}	0.016	0.016	0	0.008	0	0.009	-0.006	0.156	0.024	0	-0.016	0	0
-0.2	0.01	0.01	**1: None**	$TE_{age=60}$	0	-0.001	-0.001	0.059	0.004	-0.002	-0.002	0.062	0.004	-0.001	-0.001	0.059	0.003
				vTE_{age+1}	0	0	0	0.007	0	-0.004	-0.004	0.119	0.014	0	0	0	0
			**2: Interaction**	$TE_{age=60}$	0	0.003	0.003	0.059	0.003	0.002	0.002	0.062	0.004	0.002	0.002	0.059	0.003
				vTE_{age+1}	0.006	0.006	0	0.007	0	0.003	-0.004	0.122	0.015	0	-0.006	0	0
			**3: Both**	$TE_{age=60}$	0	0.002	0.002	0.071	0.005	-0.004	-0.004	0.102	0.01	-0.03	-0.03	0.06	0.004
				vTE_{age+1}	0.006	0.006	0	0.007	0	0.001	-0.005	0.125	0.016	0	-0.006	0	0
-0.2	0.01	0.1	**1: None**	$TE_{age=60}$	0	0	0	0.066	0.004	0	0	0.069	0.005	-0.001	-0.001	0.066	0.004
				vTE_{age+1}	0	0	0	0.007	0	-0.001	-0.001	0.141	0.02	0	0	0	0
			**2: Interaction**	$TE_{age=60}$	0	0.002	0.002	0.067	0.004	-0.001	-0.001	0.071	0.005	0	0	0.067	0.004
				vTE_{age+1}	0.006	0.006	0	0.007	0	0.007	0.001	0.142	0.02	0	-0.006	0	0
			**3: Both**	$TE_{age=60}$	0	0.005	0.005	0.078	0.006	0	0	0.119	0.014	-0.028	-0.028	0.066	0.005
				vTE_{age+1}	0.006	0.006	0	0.007	0	0.006	0	0.145	0.021	0	-0.006	0	0
-0.2	0.1	0.01	**1: None**	$TE_{age=60}$	0	0	0	0.057	0.003	-0.001	-0.001	0.058	0.003	0	0	0.056	0.003
				vTE_{age+1}	0	0	0	0.007	0	-0.005	-0.005	0.112	0.013	0	0	0	0
			**2: Interaction**	$TE_{age=60}$	0	-0.003	-0.003	0.059	0.003	-0.005	-0.005	0.062	0.004	-0.004	-0.004	0.059	0.003
				vTE_{age+1}	0.006	0.007	0	0.007	0	0.004	-0.003	0.124	0.015	0	-0.006	0	0
			**3: Both**	$TE_{age=60}$	0	-0.001	-0.001	0.069	0.005	-0.005	-0.005	0.103	0.011	-0.032	-0.032	0.057	0.004
				vTE_{age+1}	0.006	0.006	0	0.007	0	0	-0.006	0.121	0.015	0	-0.006	0	0

TE	σ	τ	Scenario	Treatment effect	True log(HR)	IPD-Poisson1 Mean	Bias	ESE	ASE	AD-Metareg Mean	Bias	ESE	ASE	AD-Netmeta Mean	Bias	ESE	ASE
-0.2	0.1	0.1	**1: None**	$TE_{age=60}$	0	0	0	0.068	0.005	0.001	0.001	0.071	0.005	0	0	0.068	0.005
				vTE_{age+1}	0	0	0	0.007	0	-0.003	-0.003	0.139	0.019	0	0	0	0
			**2: Interaction**	$TE_{age=60}$	0	0.002	0.002	0.068	0.005	0	0	0.072	0.005	0.001	0.001	0.068	0.005
				vTE_{age+1}	0.006	0.006	0	0.007	0	0.004	-0.003	0.149	0.022	0	-0.006	0	0
			**3: Both**	$TE_{age=60}$	0	0.003	0.003	0.079	0.006	0.004	0.004	0.117	0.014	-0.03	-0.03	0.068	0.005
				vTE_{age+1}	0.006	0.006	0	0.007	0	0.006	-0.001	0.144	0.021	0	-0.006	0	0

Configuration 1 represents a 3 arms network with B-C and A-C trials from which we estimate the indirect treatment effect AB, configuration 2 represent a 3 arms network with A-B, A-C and B-C trials from which we estimate the mixed (direct and indirect) treatment effect AB. AD: aggregated values, IPD: individual patient's data, ESE: empirical standard error, ASE: Average Standard Error, TE: treatment effect, σ between trial random effect for baseline risk, τ between-trial random effect for treatment effect, $TE_{age=60}$: marginal effect = log(HR) for a patient of age 60; vTE_{age+1}: the variation in log(HR) for a variation of one year of age)
