# Supplementary material for: A frequentist one-step model for a simple network meta-analysis of time-to-event data in presence of an effect modifier
Source: PLoS One. 2021 Nov 1;16(11):e0259121. doi: 10.1371/journal.pone.0259121 (PMC8559936; doi:10.1371/journal.pone.0259121)
Supplement: S1 Text — (PDF) [file pone.0259121.s007.pdf]

S1 text: Trials included in the network

The example of the current study is based on all the data of the initial MARCH study (15 trials and 6 515 patients) and 62 trials /12 129 patients out of the 87 trials / 16 485 patients of the first update of the MACH-NC study. Type of modified radiotherapy for MARCH, and timing of chemotherapy or type of loco-regional treatment were not taken into account as in the clinical studies. There is no duplication of patients as in these meta-analyses and only one hazard ratio per trial. Moreover, the two meta-analysis has been updated: update of MARCH is published (Lacas et al. Lancet Oncol, 2017 Sep;18(9)) and the one for MACH-NC is currently (December 2020) accepted for publication (Lacas Rad Oncol). Lastly, the network is simplified as compared to the one already published (Blanchard et al., J Clin Epidemiol, 2011 64(9)) and the one based on updated data that is submitted to publication. They include 6 and 16 distinct modalities of treatment respectively instead of 3 for the current study. For all this reason, its results cannot be applied to clinical practice.
